# Corrections to Be Made in the Proemium to This Volume

**Published:** 1821-03

**Authors:** 


*Corrections to be made in the* Proemium *to this Volume.*

Pages xli. and xlv. for *Dr. Gordon* read *Dr. Copland;* and add to the list of Papers referred to in page lxxxiv. "*on certain Painful Affections of the Intestinal Canal, by Dr. Powell.*"

